# A single dose of recombinant VSV-∆G-spike vaccine provides protection against SARS-CoV-2 challenge

**DOI:** 10.1038/s41467-020-20228-7

**Published:** 2020-12-16

**Authors:** Yfat Yahalom-Ronen, Hadas Tamir, Sharon Melamed, Boaz Politi, Ohad Shifman, Hagit Achdout, Einat B. Vitner, Ofir Israeli, Elad Milrot, Dana Stein, Inbar Cohen-Gihon, Shlomi Lazar, Hila Gutman, Itai Glinert, Lilach Cherry, Yaron Vagima, Shirley Lazar, Shay Weiss, Amir Ben-Shmuel, Roy Avraham, Reut Puni, Edith Lupu, Elad Bar-David, Assa Sittner, Noam Erez, Ran Zichel, Emanuelle Mamroud, Ohad Mazor, Haim Levy, Orly Laskar, Shmuel Yitzhaki, Shmuel C. Shapira, Anat Zvi, Adi Beth-Din, Nir Paran, Tomer Israely

**Affiliations:** Israel Institute for Biological Research, Ness Ziona, Israel

**Keywords:** Vaccines, SARS-CoV-2

## Abstract

The COVID-19 pandemic caused by SARS-CoV-2 imposes an urgent need for rapid development of an efficient and cost-effective vaccine, suitable for mass immunization. Here, we show the development of a replication competent recombinant VSV-∆G-spike vaccine, in which the glycoprotein of VSV is replaced by the spike protein of SARS-CoV-2. In-vitro characterization of this vaccine indicates the expression and presentation of the spike protein on the viral membrane with antigenic similarity to SARS-CoV-2. A golden Syrian hamster in-vivo model for COVID-19 is implemented. We show that a single-dose vaccination results in a rapid and potent induction of SARS-CoV-2 neutralizing antibodies. Importantly, vaccination protects hamsters against SARS-CoV-2 challenge, as demonstrated by the abrogation of body weight loss, and  alleviation of the extensive tissue damage and viral loads in lungs and nasal turbinates. Taken together, we suggest the recombinant VSV-∆G-spike as a safe, efficacious and protective vaccine against SARS-CoV-2.

## Abstract (web summary)

Here, the authors generate a replication-competent VSV based vaccine expressing SARS-CoV-2 spike protein and show protection in the hamster model with one dose. Analysis of the antibody response in mice shows induction of neutralizing antibodies and suggests a desirable Th1-biased response to the vaccine.

## Introduction

Severe acute respiratory syndrome coronavirus 2 (SARS-CoV-2), a member of the *Coronaviridae* family, is the causative agent of Coronavirus Disease 2019 (COVID-19)^1–3^. The virus was first described in late 2019 in Wuhan, China, and rapidly spread globally. Over 45 million cases worldwide were diagnosed, with over 1.1 million deaths (as of November 3, 2020, covid19.who.int).

SARS-CoV-2 is a single-stranded positive sense RNA virus decorated with the spike (S) surface glycoprotein. The S protein is a highly glycosylated type I membrane protein. The homotrimeric organization of the S protein on the viral membrane forms the typical coronaviruses S structures^4^. The S protein binds with high affinity to the angiotensin-converting enzyme 2 (ACE2) receptor. This binding induces membrane fusion and entry of the SARS-CoV-2 into host cells, thus serving as a target for neutralizing antibodies^5,6^. The SARS-CoV-2 S protein is composed of two distinct subunits, namely S1 and S2. The surface unit S1 binds the receptor, whereas the transmembrane unit S2 facilitates viral fusion to cell membranes. The S protein is activated by a cleavage at the spike S1/S2 site by host cell proteases^7^. Moreover, it has been recently shown that the SARS-CoV-2 has a newly formed furin cleavage site at the S1/S2 boundary. This novel feature dramatically affects viral entry into Vero E6 and BHK-21 cells^6^.

Vesicular stomatitis virus (VSV), a member of the *Rhabdoviridae* family, is a nonsegmented single-stranded negative sense RNA virus. VSV causes disease in animals, with a broad host range from insects to mammals. However, human VSV infection cases are rare. The VSV genome encodes for five major proteins: matrix protein (M), nucleoprotein (N), large polymerase protein (L), phosphoprotein (P), and glycoprotein (G). The L and P proteins, together with the N, form the transcriptionally active subunit of the virus. The G protein mediates both viral binding and host cell fusion with the endosomal membrane following endocytosis, and cell entry^8^.

The recombinant VSV (rVSV) platform was developed by John Rose and Michael Whitt^9,10^. rVSV was previously developed as a vaccine platform for several viral pathogens, including Ebola virus (EBOV), human immunodeficiency virus, and Crimean–Congo hemorrhagic fever virus^11,12^. As a vaccine platform, rVSV harbors several advantages: (1) The virus can be easily propagated and reach high titers, (2) it elicits strong cellular and humoral immunity in vivo, (3) elimination of the VSV-G protein, the major virulence factor of the VSV, attenuates the virus and reduces its reactogenicity, (4) VSV is sensitive to IFN-α/β, and an intact innate immune response likely restricts its replication^13^, and (5) most of the general population is seronegative for VSV^14^.

As the need for a vaccine for SARS-CoV-2 is urgent, more than 200 vaccines are being rapidly developed using a variety of technologies, including over 40 vaccines that are currently tested in clinical trials (as of November 3, 2020, https://www.who.int/publications/m/item/draft-landscape-of-covid-19-candidate-vaccines). Among them are RNA and DNA vaccines, viral vectored vaccines, recombinant proteins, live attenuated and inactivated vaccines^15^, as well as several replicating VSV-based vaccines. Currently, several of these vaccines are in advanced clinical trial phases. Here, we designed an rVSV-based vaccine (rVSV-∆G-spike), in which the VSV-G protein is replaced with the SARS-CoV-2 S protein, creating a recombinant replicating virus.

In this work, we created a cDNA vector encoding the sequence of the N, P, M, and L genes of the VSV genome, and the S protein of the SARS-CoV-2, under T7 promoter. We show that the rVSV-∆G-spike vaccine candidate is decorated by the S protein on its membrane and is expressed by infected cells. We also demonstrate that rVSV-∆G-spike is neutralized by SARS-CoV-2 convalescent serum, indicating that the S antigenicity of rVSV-∆G-spike is similar to that of SARS-CoV-2. Moreover, we have implemented a reliable experimental golden Syrian hamster model for COVID-19, enabling rVSV-∆G-spike vaccine evaluation. A single dose of the rVSV-∆G-spike vaccine candidate was found to be safe, efficacious, and provides protection against a deleterious SARS-CoV-2 challenge.

## Results

### Generation of rVSV-ΔG-spike

**Fig. 1:**
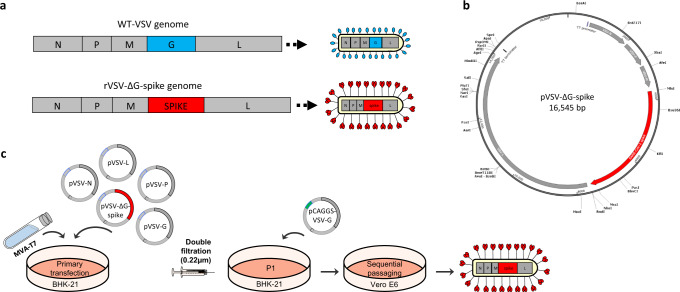
rVSV-∆G-spike design and generation strategy. **a** A schematic diagram of the genomic organization of WT-VSV (top diagram), and rVSV-∆G-spike (bottom diagram). N nucleoprotein, P phosphoprotein, M matrix, L large polymerase, G glycoprotein, SPIKE SARS-CoV-2 spike. **b** pVSV-∆G-spike map. **c** Schematic representation of the generation process of rVSV-∆G-spike vaccine. Infection of BHK-21 cells with MVA-T7, followed by cotransfection with pVSV-∆G-spike, and VSV-system accessory plasmids; transfection of BHK-21 cells with pCAGGS-VSV-G, followed by infection with the supernatant of the primary transfection, to create P1; sequential passaging in Vero E6 cells were performed creating rVSV-∆G-spike.

The goal of rapid and efficient mass vaccination calls for the implementation of replicating vaccines, such as VSV-based vaccines^14^. To this purpose, we replaced the open reading frame (ORF) of VSV-G with the full-length human codon-optimized S gene of SARS-CoV-2 (Sino Biological), within the VSV full-length expression vector, yielding pVSV-ΔG-spike (Fig. 1a, b). Primary recovery of the VSV-ΔG-spike was performed in BHK-21 cells infected with Modified Vaccinia Ankara T7 (MVA-T7), followed by cotransfection with the rVSV-ΔG-spike, and the VSV accessory plasmids encoding for VSV-N, P, L, and G proteins under control of a T7 promoter (Fig. 1c). To further support the entry of the recovered virus during the initial steps, VSV-G was expressed under a strong mammalian promoter. To remove residual MVA-T7 from the recovered virus, two sequential filtration steps were applied followed by infection of Vero E6 cells, as these cells support SARS-CoV-2 spike mediated entry, but not MVA replication. Seventy two hours post infection, the infected cells underwent syncytia formation, accompanied by significant cytopathic effect (CPE). The supernatant containing the recovered rVSV-ΔG-spike was then collected, centrifuged, and used for further passaging. Fourteen subsequent passages on Vero E6 cells were performed, aimed to eliminate the carryover of the VSV-G protein used during the initial recovery steps, and to increase the viral replication efficiency and titer.

### Characterization and analysis of rVSV-ΔG-spike

**Fig. 2:**
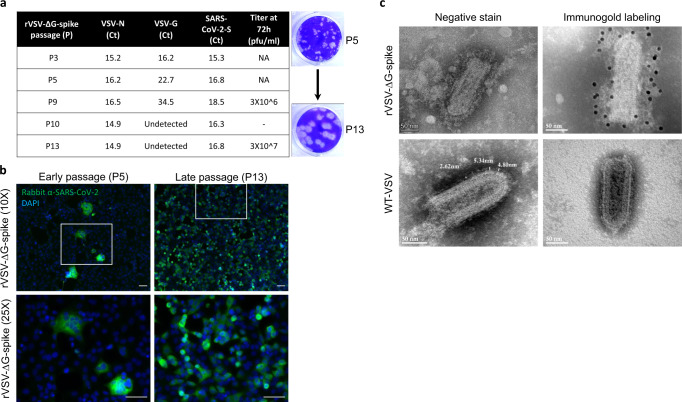
Characterization of rVSV-∆G-spike. **a** A table summarizing the genome analysis of several passages of rVSV-∆G-spike, showing elimination of VSV-G over time, together with increased titer, and formation of plaques. NA not applicable. “-” not evaluated. **b** Representative immunofluorescence images of Vero E6 cells infected with early passage (P5)-rVSV-∆G-spike, or late passage (P13)-rVSV-∆G-spike, stained with a SARS-CoV-2 antibody (green), and counterstained with DAPI for nuclei staining (blue). Bottom panels show insets at large magnification. Scale bars: 50 µm. rVSV-∆G-spike at P5 formed syncitia, whereas P13 showed individual infected cells, with no evidence of syncitia. **c** Transmission electron micrographs of P14 rVSV-∆G-spike (top panels) compared to WT-VSV (bottom panels). Right panels show immunogold labeling using gold nanoparticles conjugated antibodies directed to the spikes’ RBD. Data for **b** and **c** are representative of four and five experiments, respectively.

The above-mentioned 14 sequential passages were accompanied by morphological manifestations. The VSV-G gene carryover from the initial transfection was gradually eliminated while maintaining the SARS-CoV-2 S gene, and other VSV genomic RNA-encoded genes (represented by VSV-N gene), as demonstrated by quantitative real-time RT-PCR (Fig. 2a). The VSV-G gene was undetectable at passage 10, and thereafter.

To assess the growth kinetics of the rVSV-ΔG-spike throughout the sequential passages, rVSV-ΔG-spike titer was determined at several time points by plaque assay. During the initial passages, the effect of rVSV-ΔG-spike was characterized by the formation of large syncytia accompanied by CPE, as visualized by cell rounding, detachment, and disruption of the entire monolayer. However, traditional plaques were not observed. Plaque formation was evident only at later stages, correlating with the decrease in syncytia formation, increase in S gene expression and elimination of VSV-G gene. Accordingly, rVSV-ΔG-spike titer was increased during passaging progression, reaching 3 × 10^7^ pfu/ml, at passage 13 (P13) (Fig. 2a).

To evaluate the expression of rVSV-ΔG-spike, we performed immunofluorescence analysis using anti-SARS-CoV-2 antibodies. SARS-CoV-2 S protein was efficiently expressed in rVSV-ΔG-spike-infected Vero E6 cells (Fig. 2b). Immunostaining of rVSV-ΔG-spike infected cells displayed syncytia, mostly at the initial passages during the creation of the rVSV-ΔG-spike (P5), whereas during advanced steps (P13) showed a robust expression of the S protein in individually infected cells.

Transmission electron microscopy (TEM) was performed to analyze the ultrastructure of the rVSV-ΔG-spike. rVSV-ΔG-spike particles maintained the overall characteristic *Rhabdoviridae* bullet-shape morphology and were decorated with S protein on the particles’ membrane, already at passage 2. Passaging led to increase in the prevalence of the S structures per single particle (Fig. 2c). Furthermore, the spikes’ lengths were measured, and found to be at the range of 12–20 nm, consistent with that of the S protein on SARS-CoV-2 particles^16^. In contrast, G protein length, ranging from 2 to 6 nm, was observed on WT-VSV particles. Immunolabeling of rVSV-ΔG-spike using polyclonal antibodies directed at the receptor-binding domain (RBD) of the spike further validated the presence of S structures on the rVSV-ΔG-spike viral particle (Fig. 2c).

### S mutations acquired during the creation of rVSV-ΔG-spike vaccine candidate

The process of rVSV-ΔG-spike generation by serial passaging was accompanied by sequencing at key passages. While early in the process no significant variations were detected, the emergence of three mutations was evident at passage 9 (Table 1): (1) a synonymous mutation at position 507, (2) a nonsynonymous mutation R685G—this mutation is located at the multibasic motif of the S1/S2 cleavage site of the S protein—RRAR, thus creating a RRAG site, and (3) a nonsense mutation introducing a stop codon at position 1250 of the S protein (C1250*), leading to truncation of 24 amino acids at the cytoplasmic tail. Upon further passaging, the mutation at the S1/S2 site, as well as the stop mutation resulting in Δ24 amino acids, became dominant. Their occurrence and development, coinciding with a reduction in the VSV-G, a change in the appearance of CPE and increased viral titers suggest that these mutations may be crucial for the adaptation of rVSV-ΔG-spike to replicate in Vero E6 cells.

**Table 1:** Spike mutations acquired during serial passaging.

Mutations	Ref codon	Alt codon
P507P	CCA	CCT
R685G	AGG	GGG
C1250*	TGT	TGA

A table summarizing the mutations in the spike that were acquired during serial passaging of rVSV-∆G-spike, showing the synonymous mutation (upper row), the nonsynonymous mutation at the S1/S2 cleavage site (middle row), and the nonsense mutation introducing a stop codon leading to a truncation of 24 amino acids at the cytoplasmic tail of the S protein (bottom row). An asterisk (*) indicates stop codon.

*Ref codon* reference codon, *Alt codon* altered codon.

### Surface antigenic similarity between rVSV-ΔG-spike and SARS-CoV-2

**Fig. 3:**
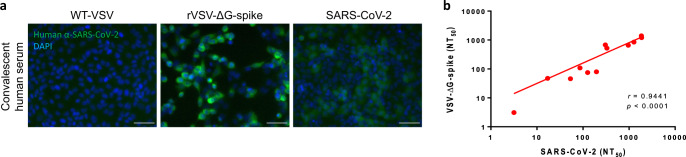
Antigenic similarity of rVSV-ΔG-spike and SARS-CoV-2. **a** Immunofluorescent images of Vero E6 cells infected with either WT-VSV (left panel), rVSV-ΔG-spike (middle panel), or SARS-CoV-2, stained with COVID-19 human convalescent serum. Representative images of five experiments are presented. Scale bars: 50 µm. **b** Correlation analysis of neutralization of rVSV-∆G-spike and SARS-CoV-2 by a panel of sera from COVID-19 convalescent patients. For each serum sample (*n* = 12), NT_50_ values were determined for neutralization of rVSV-∆G-spike or SARS-CoV-2. The NT_50_ values were plotted to determine the correlation between the neutralization assays. Spearman’s correlation *r* and *p* values are indicated. Source data are provided as a Source Data file.

We next evaluated the antigenic similarity between S structures in both SARS-CoV-2 and rVSV-ΔG-spike infected cells. Such similarity may indicate an efficient and relevant immune response elicited by rVSV-ΔG-spike. Indeed, S protein was detected by COVID-19 human convalescent sera in both rVSV-ΔG-spike and SARS-CoV-2 infected Vero E6 cells at 24 h post infection (Fig. 3a). Next, we determined the ability of several COVID-19 convalescent human sera to neutralize either rVSV-ΔG-spike, or native SARS-CoV-2, in a plaque reduction neutralization test (PRNT). The NT_50_ values, the dilution at which 50% neutralization was observed, were determined for each serum sample, for neutralization of either rVSV-ΔG-spike or SARS-CoV-2. Most tested human sera showed efficient neutralization of both rVSV-ΔG-spike and SARS-CoV-2. We observed a strong correlation (*r* = 0.944, *p* < 0.0001) between the potency of human sera to neutralize rVSV-ΔG-spike and SARS-CoV-2 (Fig. 3b). Consequently, rVSV-ΔG-spike was demonstrated as an authentic surrogate for SARS-CoV-2, and thus suitable for eliciting the desired immune response.

### rVSV-ΔG-spike vaccine efficacy in a COVID-19 hamster model

**Fig. 4:**
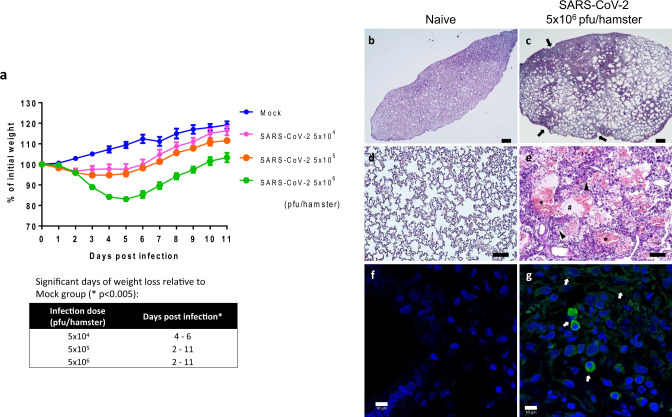
Establishment of a golden Syrian hamster SARS-CoV-2 model. **a** Body weight changes of hamsters infected with 5 × 10^4^ (*n* = 8), 5 × 10^5^ (*n* = 8), or 5 × 10^6^ (*n* = 8) pfu/hamster of SARS-CoV-2, compared to mock-infected hamsters (*n* = 4). Table shows days of significant differences, relative to mock infection. Statistical analysis was performed using one unpaired *t*-test per row, with correction for multiple comparisons using the Holm–Sidak method, *p* < 0.005. **b**–**g** Lungs were isolated and processed for paraffin embedding from naive hamsters (left panels) or SARS-CoV-2-infected hamsters (right panels, 5 × 10^6^ pfu/hamster) at 7 dpi. Sections (5 µm) were taken for H&E staining (**b**–**e**) and SARS-CoV-2 immunolabeling (**f**, **g**, nuclei-blue, SARS-CoV-2-green). **b**–**e**: scale bar = 100 µm; **f**, **g**: scale bar = 10 µm. Black arrows indicate patches of focal inflammation, pleural invagination, and alveolar collapse. An asterisk (*) indicates hemorrhagic areas. A hash symbol indicates edema and protein-rich exudates. Black arrow heads indicate pulmonary mononuclear cells. White arrows indicate SARS-CoV-2 positive immunolabeling. Naive group: *n* = 4, SARS-CoV-2 5 × 10^6^ 7 dpi group: *n* = 1. Source data are provided as a Source Data file.

To evaluate the efficacy of rVSV-ΔG-spike as a candidate vaccine against SARS-CoV-2, we established a small animal model for COVID-19 using golden Syrian hamsters (*Mesocricetus auratus*). In order to establish the hamster model, animals were infected via the intranasal route (i.n.) with SARS-CoV-2 with doses of 5 × 10^4^, 5 × 10^5^, or 5 × 10^6^ pfu and monitored for body weight changes. As shown in Fig. 4a, animals displayed weight loss of up to 3%, 5%, and 17%, respectively, in a dose-dependent manner. Days at which a statistically significant weight loss was observed are indicated below the graph. A dose of 5 × 10^6^ pfu was determined as the inoculation dose for further experiments. Also, histological sections of lungs 7 days post infection (dpi) were performed (Fig. 4b–g). Lungs of hamsters infected with 5 × 10^6^ pfu/hamster (Fig. 4c, e, g) show focal patches of inflammation, pleural invagination and alveolar collapse, large amounts of inflammatory cells infiltration, as well as hemorrhagic areas. Edema was also observed, accompanied by protein-rich exudates. Moreover, immunostaining of the infected lung with an anti-RBD rabbit polyclonal antibody showed presence of SARS-CoV-2 positive cells (Fig. 4g) as compared to naive hamsters’ lungs (Fig. 4f).

**Fig. 5:**
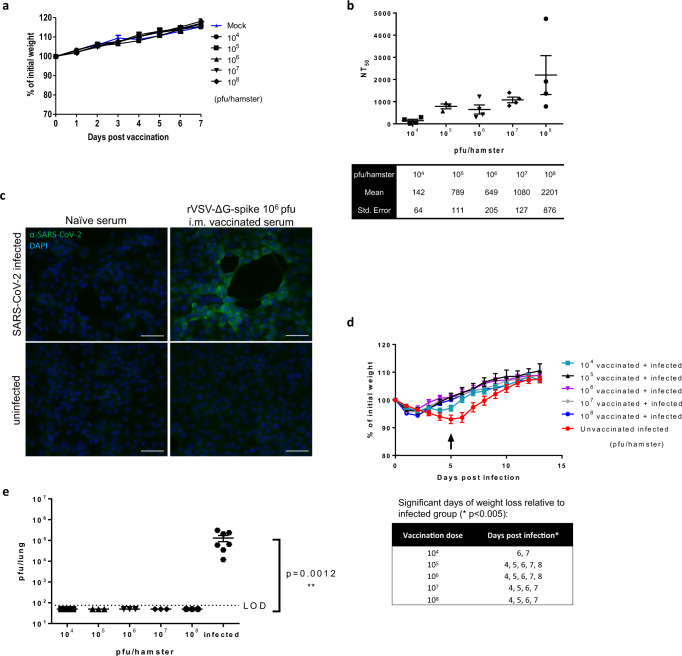
A single-dose i.m. rVSV-∆G-spike vaccine safety and efficacy in hamsters following SARS-CoV-2 challenge. **a** Body weight changes of mock-vaccinated hamsters (*n* = 4), and hamsters vaccinated with rVSV-∆G-spike ranging from 10^4^ to 10^8^ pfu/hamster (*n* = 10, *n* = 11, *n* = 11, *n* = 11, *n* = 10, for each vaccinated group, respectively). **b** NT_50_ values of i.m. vaccinated hamsters’ sera (10^4^–10^8^ pfu/hamster) against SARS-CoV-2 (*n* = 4 for 10^4^, 10^6^, 10^7^, and 10^8^, *n* = 3 for 10^5^). Means and SEM are indicated below the graph. **c** Representative immunofluorescence images of Vero E6 cells infected with SARS-CoV-2 (upper panels) or uninfected (lower panels), labeled with serum from either naive (left panel) or rVSV-∆G-spike (10^6^ pfu/hamster) i.m. vaccinated hamsters (right panel). Representative images of three experiments are presented. Scale bars: 50 µm. **d** Body weight changes of hamsters infected with SARS-CoV-2, and hamsters vaccinated with 10^4^–10^8^ pfu/hamster and infected with 5 × 10^6^ pfu/hamster 25 days post vaccination. Arrow indicates 5 dpi—hamsters were sacrificed and lungs were removed for viral load. For vaccinated groups 10^4^–10^7^: days 0–5 (*n* = 12), days 6–12 (*n* = 9), 10^8^ group: days 0–5 (*n* = 11), day 6–12 (n = 8). For unvaccinated infected: days 0–5 (*n* = 14), days 6–12 (*n* = 12). Statistical significance was determined using two-tailed one unpaired *t*-test per row, with correction for multiple comparisons using Holm–Sidak method. *p* < 0.005. **e** Viral loads in lungs (5 dpi) of hamsters infected with 5 × 10^6^ pfu/hamster of SARS-CoV-2 (*n* = 7), and hamsters vaccinated with 10^4^–10^8^ pfu/hamster of rVSV-∆G-spike, and then infected with 5 × 10^6^ pfu/hamster of SARS-CoV-2 (*n* = 3 for each vaccinated group). Limit of detection (LOD) 75 pfu/lung. All vaccinated groups show statistical significance, compared to infected unvaccinated group as determined by one-way ANOVA nonparametric Kruskal–Wallis test, with Dunn’s multiple comparisons test, *p = 0.0012. Data for **a**, **d**, and **e** are presented as mean values ± SEM. Source data are provided as a Source Data file.

Next, we examined the safety and efficacy of a single dose of rVSV-ΔG-spike vaccine in the hamster model. To that end, hamsters were vaccinated by intramuscular (i.m.) injection at increasing doses of rVSV-ΔG-spike: 10^4^, 10^5^, 10^6^, 10^7^, or 10^8^ pfu, and compared to mock-vaccinated hamsters. Following vaccination, animals were monitored daily for body weight changes and morbidity. No signs of lesion were observed at the site of injection (not shown). As seen in Fig. 5a, animals in all groups gained weight and did not show any signs of morbidity, suggesting that rVSV-ΔG-spike is safe at the tested doses. The efficacy of the vaccine was evaluated for the ability of a single vaccination of each dose to elicit neutralizing antibodies against SARS-CoV-2 by PRNT. All tested vaccine doses induced a neutralization response in a dose-dependent manner following a single-dose vaccination (Fig. 5b). The binding capacity of the induced antibodies is demonstrated by immunofluorescent staining of Vero E6 cells infected with SARS-CoV-2, by using sera from 10^6^ pfu-vaccinated hamsters (Fig. 5c). To evaluate the efficacy of the rVSV-ΔG-spike vaccine to protect against SARS-CoV-2, vaccinated hamsters were challenged by i.n. instillation with 5 × 10^6^ pfu of SARS-CoV-2 per animal (~4 weeks post vaccination). Following challenge, unvaccinated animals were morbid, exhibiting a gradual weight loss up to 5 dpi. In contrast, rVSV-ΔG-spike vaccinated hamsters showed a mild weight loss immediately after infection, followed by a recovery, amounting to a significant improvement in body weight at 4–7 dpi for all doses, except the lowest vaccination dose of 10^4^ pfu at which a significant improvement was detected at 6 and 7 dpi (Fig. 5d).

### Efficacy and viral load analysis of rVSV-ΔG-spike vaccinated hamsters

Five days following challenge with SARS-CoV-2, lungs were removed for viral load analysis. The presence of infectious SARS-CoV-2 was detected in infected unvaccinated lungs, with an average viral titers of 1.3 × 10^5^ pfu/lung (*n* = 7), whereas viral titers in lungs of all doses of vaccinated and infected animals (10^4^–10^8^ pfu, *n* = 3 for each vaccination dose) were below the limit of detection (LOD, 75 pfu/lung) (Fig. 5e).

**Fig. 6:**
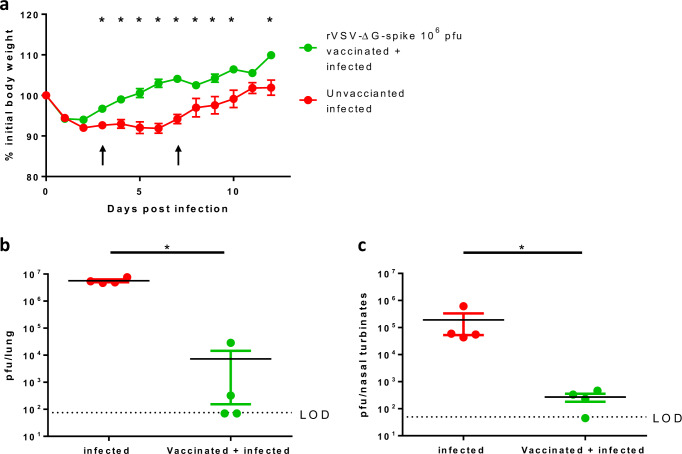
Viral load analysis in organs of rVSV-∆G-spike vaccinated hamsters following SARS-CoV-2 challenge. **a** Body weight changes of SARS-CoV-2 infected hamsters (5 × 10^6^ pfu) 25 days post vaccination with 1 × 10^6^ pfu, or unvaccinated. Arrows indicate 3 and 7 dpi—hamsters were sacrificed. Number of animals per group: days 0–3 *n* = 15, days 4–7 *n* = 7, days 8–12 *n* = 3. Statistical significance was performed by using two-tailed one unpaired *t*-test per row, with correction for multiple comparisons using Holm–Sidak method, *p < 0.005. Viral loads in lungs (**b**) and nasal turbinates (**c**) at 3 dpi of hamsters infected with SARS-CoV-2 (5 × 10^6^ pfu/hamster, *n* = 4), and hamsters vaccinated with rVSV-∆G-spike (10^6^ pfu/hamster), followed by infection with SARS-CoV-2 (5 × 10^6^ pfu/hamster, *n* = 4). LOD: 75 pfu/lung, 50 pfu/nasal turbinates. Data for **a**–**c** are presented as mean values ± SEM. Significance analysis for **b** and **c** was performed using two-tailed unpaired Mann–Whitney nonparametric test, *p = 0.0286. Source data are provided as a Source Data file.

All tested vaccination doses were shown to be safe and efficacious. Notably, doses of 10^5^ and 10^6^ pfu/hamster elicited a significant and prolonged protection window at 4–8 dpi, and 10^6^ was chosen for further meticulous evaluation. Hamsters were vaccinated with a single dose of 10^6^ pfu, followed by i.n. instillation of 5 × 10^6^ pfu SARS-CoV-2, and monitored for body weight changes for 12 dpi. The protective effect of the vaccine was demonstrated by a significant difference in body weight loss starting at 3 dpi and onward (Fig. 6a).

It was previously shown that at days 2–3 post SARS-CoV-2 infection, lungs, trachea, and nasal turbinates display high viral titers, accompanied by extensive tissue damage^17–19^. To that end, lungs and nasal turbinates were removed and analyzed for viral load at 3 dpi. Lungs extracted from infected hamsters showed average viral titers of 5.6 × 10^6^ pfu/lung (*n* = 4), whereas viral titers in lungs of vaccinated and infected animals were significantly lower (average viral titers of 7.3 × 10^3^ pfu/lung) (*n* = 4) (Fig. 6b). In addition, nasal turbinates of infected hamsters showed average viral titers of 1.9 × 10^5^ pfu/nasal turbinates (*n* = 4), whereas viral titers in nasal turbinates of vaccinated and infected animals similarly showed a significant reduction in average viral titers to 2.8 × 10^2^ pfu/nasal turbinates (*n* = 4) (Fig. 6c). Taken together, a single-dose vaccination of hamsters was able to reduce the viral titer by three orders of magnitude, in both tested organs.

### Histopathological analysis of rVSV-ΔG-spike vaccinated lungs

**Fig. 7:**
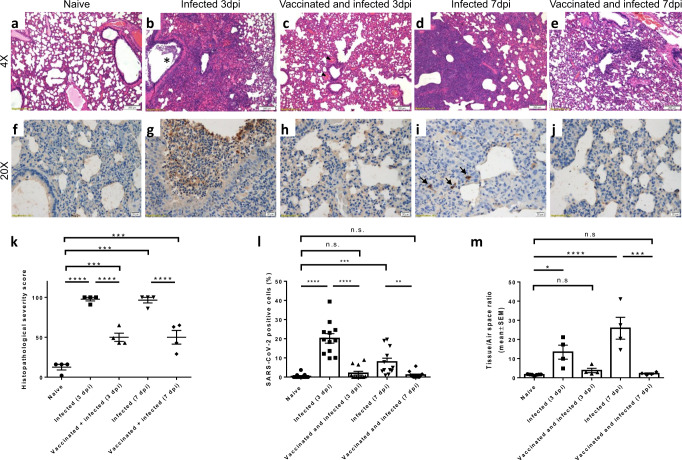
Histopathological analysis of rVSV-∆G-spike i.m. vaccinated and infected hamsters’ lungs at 3 and 7 dpi. General histology (H&E) and SARS-CoV-2 DAB immunolabeling of naive, unvaccinated infected (5 × 10^6^), and vaccinated (10^6^ pfu) hamsters’ lungs, at 3 and 7 dpi. Lungs were isolated and processed for paraffin embedding from naive (**a**, **f**), infected (5 × 10^6^ pfu) (**b**, **g** for 3 dpi and **d**, **i** for 7 dpi) and vaccinated and infected (**c**, **h** for 3 dpi and **e**, **j** for 7 dpi). Sections (4 µm) were taken for H&E staining (**a**–**e**) and SARS-CoV-2 DAB immunolabeling (**f**–**j**, positive SARS-CoV-2—brown, hematoxylin counterstaining—blue). An asterisk (*) indicates cellular debris in bronchiolar lumen. Black arrow heads indicate congestion of blood in blood vessels. Black arrows indicate positive stained cells. **a**–**e**: scale bar = 200 µm; **f**–**j**: scale bar = 20 µm. **k** Histopathological severity analysis of hamsters lungs of naive, vaccinated, and infected lungs, at 3 and 7 dpi. **l** Digital morphometric analysis of DAB immunohistochemical staining for SARS-CoV-2 in lungs of naive, infected, and vaccinated lungs, at 3 and 7 dpi. **m** Tissue/air space analysis of naive, infected, and vaccinated lungs, at 3 and 7 dpi. Data for **a**–**j** was taken for five groups. Each group includes four animals. For each animal, five fields were imaged and analyzed. Data for **k**–**m** are presented as mean values ± SEM. Statistical analyses for **k**–**m** were performed by one-way ANOVA with Tukey’s multiple comparisons test, with *p* < 0.0001, *p* = 0.0006, *p* < 0.0001, respectively. Source data are provided as a Source Data file.

To further characterize the efficacy of the rVSV-ΔG-spike, a detailed histopathological evaluation was performed on lungs of naive, infected, and vaccinated and infected hamsters at 3 and 7 dpi. Naive animals showed no lesions and served as a negative control reference (Fig. 7a, f). The lungs of infected animals showed signs of viral pneumonia, characterized by severe necrosis and inflammation. The interstitium and alveoli were infiltrated mostly by neutrophils, and fibrin deposition was evident in the severe cases. The number of lymphocytes involved was relatively low. Necrosis was found in the alveolar structures of the infected hamsters. Vasodilatation and congestion of the interstitial blood vessels was also noted. The bronchial epithelium was relatively intact, yet the submucosa was often infiltrated by lymphocytes and to some extent, cellular debris were observed in the bronchioles’ lumen. The bronchial epithelium of the infected hamsters’ lungs remained relatively unaffected and the submucosa was often infiltrated by lymphocytes. The distribution pattern of the infection in the infected lung is multifocal to coalescing. Some cellular debris were spotted in the bronchiolar lumen (Fig. 7b, d). Vaccination alleviated the disease manifestations exhibiting significantly milder form of pneumonia following infection, compared to the unvaccinated infected hamsters. Infiltration of neutrophils, hyperemia, and congestion of the lung interstitium and necrotic areas were occasionally observed, yet in a milder severity compared to the infected unvaccinated group (Fig. 7c, e). Quantitative analysis of the lung manifestations severity was performed on samples 3 and 7 dpi using a severity scoring scale according the American Thoracic Society Documents^20^. Based on presence of neutrophils in alveoli and in interstitium, fibrin, cellular debris, and thickened alveolar septa, a total score was summed for each animal in all tested groups, and histopathological severity score was determined. A significant damage was shown for all infected unvaccinated hamsters. For vaccinated hamsters following infection, a significant protection of the lungs was shown, displaying a reduced severity in lung damage, at both 3 and 7 dpi (Fig. 7k). 3,3′-diaminobenzidine (DAB) staining was performed to evaluate the presence of SARS-CoV-2 antigens in infected and vaccinated hamsters at both 3 and 7 dpi. Positive SARS-CoV-2 cells were detected in infected lungs of both 3 and 7 dpi, with an average of 20.16% (3 dpi) and 7.87% (7 dpi), whereas vaccinated hamsters’ lungs showed no, or low SARS-CoV-2 staining, with an average of 2%, and 1.05% positive cells, at 3 and 7 dpi, respectively, (Fig. 7l). These results were further supported by tissue/air space analysis demonstrating significant increase in tissue/air space ratio in infected lungs at both 3 and 7 dpi compared to the naive lungs (Fig. 7m), and a significant reduction of tissue/air ratio between infected and immunized lungs at both 3 and 7 dpi. Vaccinated lungs at both time points reached the baseline ratio similar to that of naive samples (Fig. 7m). Similar results for antibody binding, safety, neutralization, and protection (Supplementary Figs. 1 and 2) were obtained by s.c. vaccination with 10^6^ pfu/hamster), further supporting rVSV-ΔG-spike as a potential vaccine.

### Antibody isotype profile induced by rVSV-ΔG-spike vaccination

**Fig. 8:**
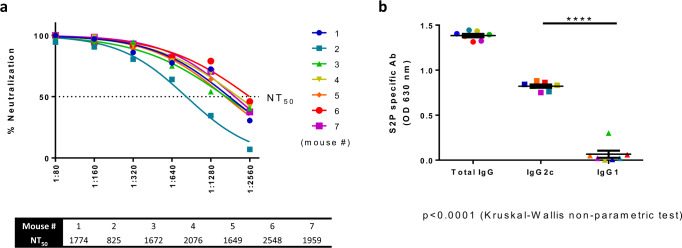
Th1 and Th2 isotytpe analysis of rVSV-∆G-spike induced antibodies. rVSV-∆G-spike vaccinated (10^7^ pfu/mouse, *n* = 7) C57BL/6J mice sera analysis for **a** NT_50_ values against SARS-CoV-2 as determined by PRNT, and **b** levels of S2P specific binding antibodies: total IgG, IgG2c, and IgG1 as determined by ELISA. Statistical significance was determined using one-way ANOVA nonparametric test, with Kruskal–Wallis test: ****p < 0.0001. Each mouse is represented by a different color. Source data are provided as a Source Data file.

The Th1/Th2 profile following vaccination is of major importance, and the aspired induced antibody profile is of a Th1 response. To that end, we evaluated the balance of Th1 and Th2 response to the rVSV-ΔG-spike vaccine through the differential induction of antibody isotypes. Due to the lack of suitable reagents for hamster isotyping, we performed isotype profiling in mice. C57BL/6J mice were vaccinated i.m. with 10^7^ pfu/mouse. Vaccinated mice sera were analyzed 14 days following vaccination for both neutralizing antibodies and S antigen-specific total IgG, as well as IgG2c and IgG1 isotypes, as surrogates of Th1 and Th2 responses, respectively. Vaccination elicited high levels of neutralizing antibodies in all mice, with NT_50_ values at the range of 825–2548 titers (Fig. 8a). High levels of IgG2c were observed in all vaccinated mice, as opposed to low levels of IgG1 (Fig. 8b), indicating the induction of a desirable and safe Th1-biased response to the rVSV-ΔG-spike vaccine.

## Discussion

The urgent need for an efficacious SARS-CoV-2 vaccine set us to develop a rVSV-ΔG-spike-based vaccine. Considering the requirements for induction of rapid and effective immune response, efficient production, and a strong safety profile, we chose the rVSV platform. The resulting rVSV-ΔG-spike vaccine has the following attributes: (1) it efficiently replicates in cultured cells, (2) the spike is robustly expressed both on the viral surface and in infected cells, (3) it induces antibodies that effectively bind and neutralize the infectious SARS-CoV-2 virus, and induce a Th1-biased immune response, and (4) the vaccine proved effective in the golden Syrian hamster model.

We show here that the rVSV-ΔG-spike vaccine is safe, well tolerated, elicits antibodies, able to bind and neutralize SARS-CoV-2 efficiently, and offers protection from high-dose SARS-CoV-2 challenge in concordance with viral clearance and alleviation of the disease manifestations. Conversely, unimmunized hamsters display rapid deterioration, significant weight loss, as well as significant viral load in both nasal turbinates and lungs, accompanied by tissue damage.

The rVSV-ΔG-spike possess several features that contribute to its safety potential as a vaccine candidate. VSV-G is considered a major virulent factor of VSV, and its elimination is known to serve as an attenuating factor^14^. Moreover, removal of the G gene from the VSV genome, together with the expression and presentation of the S protein, restricts the viral entry only to cells expressing the ACE2 receptor, further significantly contributing to its safety profile^6^. Also, VSV is sensitive to IFN-α/β, and an intact innate immune response likely controls VSV replication^13^, thus contributing to the safety of rVSV-ΔG platform.

Immunofluorescence and electron microscopy analyses demonstrated S protein expression, its membrane incorporation, and similar structural organization of the spike of the rVSV-ΔG-spike vaccine candidate to that of SARS-CoV-2 on viral particles. Preservation of the antigenicity of the S protein was further demonstrated by the high correlation of the neutralization capacity of a variety of human convalescent sera to neutralize both the rVSV-ΔG-spike as well as SARS-CoV-2.

Considering the need for vaccine mass production, a platform able to achieve high titers in cultured cells is preferable. During establishment of the rVSV-ΔG-spike vaccine, the observed phenotypic changes, namely adequate S expression and organization, reduced syncytia, and increased CPE, occurred concomitantly with a gradual increase in the rVSV-ΔG-spike viral titer, reaching titers potentially suitable for human vaccination, as previously shown for rVSV-EBOV^14^.

During the development of the rVSV-ΔG-spike vaccine, three unique mutations emerged, including the C1250* mutation leading to a truncation of 24 amino acids at the cytoplasmic tail of the S protein. The cytoplasmic tail of both SARS-CoV-1 and SARS-CoV-2 has been associated with the assembly of the virus and with its infectivity. This cytoplasmic tail possesses an ER retention signal^21^. Data from several studies on both SARS-CoV-1 that emerged in 2003 and the current SARS-CoV-2 reported cytoplasmic tail truncations, either deliberately or naturally occurring. These studies described the advantage of such truncations in the virus assembly, organization, and infectivity. As such, a cytoplasmic tail deletion of 19 amino acids in spike of SARS-CoV-1 allowed efficient assembly and incorporation of the spike into VSV particles, resulting in high titers of VSV-SARS-St19^22,23^. A similar deletion of 19 amino acids in SARS-CoV-2 spike was also shown to enhance expression of SARS-CoV-2 in mammalian cells^24^. Moreover, several recent studies establishing pseudotyped SARS-CoV-2 BSL2 platforms reported the occurrence of one or two independent stop mutations, leading to 21 or 24 amino acids truncations in the cytoplasmic tail of SARS-CoV-2 spike^25,26^.

The nonsynonymous mutation R685G generated during passaging of the rVSV-ΔG-spike led to a change in the S1/S2 motif RRAR, creating a RRAG site. It was shown that the S1/S2 site of the S protein contains a multibasic motif which is processed by the host cell furin protease. This site is essential for cell–cell fusion, and was shown to mediate infection of human lung cells^7^. Additional study^27^ demonstrated that a variant of SARS-CoV-2 with a deletion in the furin site has reduced virulence in hamsters, further substantiating the important role of this RRAR motif in virus infection and pathogenesis. This suggests that the R685G mutation in the rVSV-ΔG-spike may contribute to its attenuation.

Taken together, our data suggest that both the stop mutation leading to Δ24, and the S1/S2 cleavage site mutation of the rVSV-ΔG-spike vaccine candidate contribute to S protein expression, efficient assembly of the viral particle and its ability to replicate and propagate in Vero E6 cells, thus improving its stability, attenuating its virulence, and contributing to the overall safety of this candidate vaccine.

Vaccination of hamsters via the i.m. route resulted in a dose-dependent induction of neutralizing antibodies against SARS-CoV-2, thus providing a reliable tool for estimating vaccine efficacy. Notably, neutralizing antibodies titers induced by 10^6^ pfu/hamster administered s.c. were lower, as compared to the titers induced by i.m. injection. The differences between i.m. and s.c. vaccination were previously explored, and though the i.m. and s.c. drain to different lymph nodes, there were no differences in the magnitude or quality of antigen-specific cellular and humoral responses over time^28^. However, for several other vaccines such as hepatitis B, rabies, and influenza vaccines, it was demonstrated that in contrast to i.m., the s.c. injection may result in slow mobilization and antigen processing, and impair vaccine efficacy^29^. It may also lead to a lower level of antibody responses^30^. Though i.m. and s.c. seem similar and comparable, and based on the clinical experience with VSV-EBOV vaccination intramuscularly, we chose the i.m. injection as the vaccine route of choice.

Previous works presented the golden Syrian hamster as a model for SARS-CoV-2 pathogenesis and transmission^17,31^. In this work, we implemented the golden Syrian hamster model to evaluate the rVSV-ΔG-spike vaccine safety and efficacy. The hamster model is a robust and reproducible model as evident by the dose-dependent response in body weight to different SARS-CoV-2 infection doses. Vaccination of hamsters with rVSV-ΔG-spike i.m., and also s.c., prior to inoculation with SARS-CoV-2 provided protection, as demonstrated by minimal weight loss, followed by a remarkable recovery within a few days and a complete return to baseline by 5 or 6 dpi. This is in contrast with unvaccinated hamsters, which displayed prolonged disease peaking at day 5, followed by a slower and gradual recovery.

It was previously shown that viral titers of SARS-CoV-2 infected hamsters reach a peak at 3 dpi in the nasal turbinates, trachea and lungs, and gradually decrease^18^. The viral load of SARS-CoV-2 in the lungs of hamsters vaccinated with rVSV-ΔG-spike was analyzed at 3 and 5 dpi. At both time points, infectious virus titers in lungs of infected hamsters were high, whereas in vaccinated hamsters a significant reduction in infectious virus was observed at 3 dpi, and was eliminated completely at 5 dpi. A 3 log reduction in viral titer was also observed in nasal turbinates of vaccinated hamsters at 3 dpi in comparison to infected unvaccinated hamsters. Importantly, histopathological analysis of lungs extracted at 3 and 7 dpi from vaccinated animals showed minimal pathology, reduced tissue/air ratio, and low number of SARS-CoV-2 positive cells, as opposed to SARS-CoV-2 infected hamsters, exhibiting extensive damage and a strong SARS-CoV-2 signal. Taken together, we show a significant recovery of rVSV-ΔG-spike vaccinated hamsters, as demonstrated by protection, reduced damage, and minimal viral antigen by 3 and 7 dpi, similar to that of naive hamsters’ lungs.

A balanced Th1 and Th2 response is crucial for vaccine safety. Vaccine-associated enhanced respiratory disease was previously reported as a deleterious vaccine outcome (for vaccines such as respiratory syncytial virus), and was associated with Th2-biased immune response^32^. In order to evaluate the nature of the immune response to rVSV-ΔG-spike vaccine, and due to the lack of suitable reagents for hamsters, we performed antibody isotype analysis in rVSV-ΔG-spike-vaccinated mice. All vaccinated mice elicited high titers of neutralizing antibodies. Evaluation of S protein specific IgG2c and IgG1, as surrogates for Th1 and Th2, respectively, showed a significantly higher levels of IgG2c indicating a profound Th1 response in all vaccinated mice, further supporting the data presented so far on the efficacy and safety of the vaccine.

Clinical trials with rVSV-EBOV were conducted since 2014 in various locations worldwide and the efficacy and safety of the vaccine were tested at multiple doses ranging from 3 × 10^5^ to 3 × 10^8^ pfu. Vaccination with rVSV-EBOV was safe, with reports of transient and mild adverse events such as injection-site pain, headache, fatigue, myalgia, fever, chills, skin rash, and arthralgia^12^. At the end of 2019, rVSV-EBOV (under the trade name ERVEBO^®^) was approved by the FDA for the prevention of Zaire ebolavirus-caused disease in individuals 18 years of age and older (fda.gov), and is administered at 1 ml of at least 7.2 × 10^7^ pfu. Based on this experience, we expect rVSV-ΔG-spike vaccine to demonstrate similar safety and efficacy in humans.

Many vaccine candidates for SARS-CoV-2 are currently being developed, including inactivated SARS-CoV-2 based vaccines, protein-, DNA- and RNA-based vaccines, as well as replicating viral vectors. Among the viral vectors are few several adenovirus-, flavi-, measles- horsepox-, and VSV-based vaccines (https://www.who.int/publications/m/item/draft-landscape-of-covid-19-candidate-vaccines). Such VSV-based SARS-CoV-2 vaccine is being developed by Merck, and is currently at preclinical stages. Also, a recent work demonstrated the induction of efficacious and protective immunity in mice using VSV-eGFP-SARS-CoV-2 vector^33^. Our data showing induction of neutralizing antibodies and favored induction of IgG2c isotypes by the vaccine are in agreement with that study, strongly supporting the efficacy and safety of rVSV-ΔG-spike based vaccine candidates.

In conclusion, we generated rVSV-ΔG-spike, a recombinant replication-competent VSV-based vaccine candidate expressing the SARS-CoV-2 S protein. The rVSV-ΔG-spike resembles the SARS-CoV-2 in spike expression properties, antigenicity, and ability to induce neutralizing, Th1-favored antibodies. Moreover, a single-dose vaccination of hamsters with rVSV-ΔG-spike elicited a safe, effective, and sufficient neutralizing antibody response against SARS-CoV-2 challenge. The vaccination provided protection against SARS-CoV-2 inoculation, as manifested by reduced morbidity, lung protection, and rapid viral clearance. These results pave the way for further examination of rVSV-ΔG-spike in clinical trials as a vaccine against SARS-CoV-2.

## Methods

### Cell lines and viruses

Baby hamster kidney cells (BHK-21, ATCC^®^ CCL-10) and African green monkey kidney clone E6 cells (Vero E6, ATCC^®^ CRL-1586™) were grown in growth medium [Dulbecco’s Modified Eagle’s Medium (DMEM) containing 10% fetal bovine serum (FBS), MEM nonessential amino acids (NEAA), 2 mM l-glutamine, 100 Units/ml penicillin, 0.1 mg/ml streptomycin, 12.5 Units/ml nystatin (P/S/N), all from Biological Industries, Israel]. Cells were cultured at 37 °C, 5% CO_2_ with 95% humidity.

SARS-CoV-2 (GISAID accession EPI_ISL_406862) was kindly provided by Bundeswehr Institute of Microbiology, Munich, Germany. Virus stocks were propagated (four passages) and titered on Vero E6 cells. Handling and working with SARS-CoV-2 virus were conducted in a BSL3 facility in accordance with the biosafety guidelines of the Israel Institute for Biological Research (IIBR). VSV-Indiana (WT-VSV) was kindly provided by Prof. Eran Bacharach (Tel Aviv University).

### Plasmid construction

The pVSV-spike expression plasmid was constructed by PCR amplification of the full-length human codon-optimized S gene from pCMV3-SARS-CoV-2 S expression plasmid (Sino Biological, Cat# VG40588-UT) using the following primers: forward –atcgatctgtttacgcgtcactATGTTTGTGTTCCTGGTGCTGC; reverse – atgaagaatctggctagcaggatttgagTCAGGTGTAGTGCAGTTTCACTCC. The amplified PCR product was digested by *MluI* and *NheI* restriction enzymes (NEB), and was ligated into the pVSV-FL+(2) vector (Kerafast), precut by the same enzymes to remove the VSV-G gene. The ligated plasmid was electroporated into DH5α electro-competent cells, and selected by ampicillin resistance.

### Recovery of rVSV-ΔG-spike

BHK-21 cells were infected with MVA-T7 virus for 1 h, followed by cotransfection of five plasmids: the full-length rVSV-ΔG-spike (described above), together with the VSV accessory plasmids encoding for VSV-N, P, L, and G proteins (Kerafast), all of which were under T7 promoter control. The primary transfection was performed using calcium phosphate method. BHK-21 cells were transfected with pCAGGS-VSV-G plasmid to assist in creating passage 1 (P1). Forty-eight hours following primary transfection, the supernatant containing the recovered VSV-spike was collected, centrifuged at 1300 × *g* × 5 min to remove cell debris, and filtered twice using 0.22 µM filter to remove residual MVA-T7 virus. pCAGGS-VSV-G transfected BHK-21 cells were then infected with the total amount of the filtered supernatant. Seventy-two hours post infection the supernatant was collected, centrifuged, and used for sequential passaging in Vero E6 cells. rVSV-ΔG-spike was propagated in DMEM containing 5% FBS, MEM NEAA, 2 mM l-glutamine, and P/S/N. Fifteen millimolar D-Trehalose was added to the rVSV-ΔG-spike prior to storage at −80 °C.

### Viral titration

Vero E6 cells were seeded in 12-well plates (5 × 10^5^ cells/well) and grown overnight in growth medium. Serial dilutions of rVSV-ΔG-spike were prepared in infection medium (MEM containing 2% FBS with NEAA, glutamine, and P/S/N), and used to infect Vero E6 monolayers in duplicates or triplicates (200 µl/well). Plates were incubated for 1 h at 37 °C to allow viral adsorption. Then, 2 ml/well of overlay [MEM containing 2% FBS and 0.4% tragacanth (Merck, Israel)] was added to each well and plates were incubated at 37 °C, 5% CO_2_ for 72 h. The media were then aspirated, and the cells were fixed and stained with 1 ml/well of crystal violet solution (Biological Industries, Israel). The number of plaques in each well was determined, and rVSV-ΔG-spike titer was calculated.

### Plaque reduction neutralization test

Vero E6 cells were seeded in 12-well plates as described above. Sera from 12 convalescent COVID-19 patients was collected by the National Blood Services of “Magen David Adom” in Israel within a protocol for plasma donation. All convalescent volunteers gave their informed consent to the National Blood services of Magen David Adom. The study was approved by the ethics committee of the Israeli Ministry of Health (0083-20-WOMC)^34^. We have complied with all relevant ethical regulations for work with human samples. Additional sera used: rVSV-ΔG-spike vaccinated hamsters’ sera, SARS-CoV-2 infected hamsters’ sera, and mock-infected hamsters’ sera. All sera were heat-inactivated (HI) (at 56 °C or 60 °C for 30 min), then diluted in twofold serial dilutions (between 1:20 and 1:40,960) in 400 µl of infection medium, mixed with 400 µl of either 300 pfu/ml of rVSV-ΔG-spike or SARS-CoV-2, and incubated at 37 °C, 5% CO_2_ for 1 h. Monolayers were washed once with DMEM w/o FBS (for SARS-CoV-2 neutralization only) and 200 µl of each serum–virus mixture was added in triplicates to the cells for 1 h at 37 °C. Virus mixture without serum served as control. Two milliliters per well overlay were added to each well and plates were incubated at 37 °C 5% CO_2_ for 48 (for SARS-CoV-2) or 72 h (for rVSV-ΔG-spike). Following incubation, overlay was aspirated and the cells were fixed and stained with 1 ml/well of crystal violet solution. The number of plaques in each well was determined, and the serum dilution that neutralizes 50% of the virions (NT_50_) was calculated using Prism software (GraphPad Software Inc.).

### Immunofluorescence analysis

Overall, 1.5 × 10^5^ Vero E6 cells were seeded in a eight-well chamber (LabTek™, Nunc) and infected with WT-VSV, SARS-CoV-2, or rVSV-ΔG-spike for 24 h. Cells were then fixed with 3% paraformaldehyde (PFA) in PBS for 20 min and permeabilized with 0.5% Triton X-100 for 2 min. The fixed cells were blocked with PBS containing 2% FBS and stained with either hyperimmune rabbit serum from intravenous SARS-CoV-2 infected rabbits diluted 1:200 (in-house preparation), naive or vaccinated hamster sera diluted 1:200, or COVID-19 convalescent human sera diluted 1:200 for 1 h. After washing with PBS, cells were incubated with either Alexa Fluor 488 conjugated goat anti-rabbit at a dilution of 1:200 (Sigma, Israel, Cat# F6005), Alexa Fluor 488 conjugated goat anti-hamster at a dilution of 1:100 (Jackson, Cat# 107-545-142), or Alexa Fluor 488 conjugated goat anti-human at a dilution of 1:200 (Sigma, Cat# F0132, lot SLBR4951V). Nuclei were visualized by 4′,6-diamidino-2-phenylindole (DAPI). Images were acquired by an Axioskop (Zeiss) equipped with a DS-iR1 camera and NIS-elements software (Nikon).

### Ultrastructure analysis

To determine the ultrastructure of the rVSV-ΔG-spike or WT-VSV, TEM was performed. Carbon-coated grids were immersed in double distilled water (DDW). Clarified supernatants were absorbed to the grids by a drop-on-grid method for 15–20 min. Filtered blocking solution of 2% FBS in PBS was added to the grids for 20 min. Immunogold labeling was performed using polyclonal antibody (pAB RBD SBF40150-T62) directed at the RBD of spike at a dilution of 1:30, followed by washing (×3) with PBS and then labeling with gold-conjugated goat anti-rabbit secondary antibody (Sigma, G3779) at a dilution of 1:20. The grids were washed with PBS (×3) and DDW (twice). Grids were stained with 1% phosphotungstic acid and examined with a Tecnai T12 TEM [Thermo Fisher Scientific formerly FEI)] operated at 120 kV and equipped with a Gatan ES500W Erlangshen camera.

### Real-time RT-PCR

RNA was extracted by Viral RNA mini kit (Qiagen, Germany) as per the manufacturer’s instructions. Real-time RT-PCR was performed using the SensiFAST^TM^ Probe Lo-ROX one-step kit (Bioline). In each reaction, the primers final concentration was 600 nM and the probe concentration was 300 nM. Primers and probes were designed using the Primer Express Software (Applied Biosystems) and purchased from Integrated DNA Technologies, Inc. Probes were ordered as 6-FAM and ZEN/Iowa Black FQ combination. The primers and probes used: N gene: forward: TGATCGACTTTGGATTGTCTTCTAA, reverse: TCTGGTGGATCTGAGCAGAAGAG, probe: ATATTCTTCCGTCAAAAACCCTGCCTTCCA; G gene: forward: ATTGCCCGTCAAGCTCAGAT, reverse: CCGTCTGCTTGAATAGCCTTGT, probe: CACAGCCTTACAAGTCAAAATGCCCAAGA; S gene: forward: GAGTGAGTGTGTGCTGGGACAA, reverse: AAACACTCCCTCCCTTGGAAA, probe: AGTTTTCCACAGTCTGCCCCTCATGGA.

### Whole-genome sequencing and data analysis

The SMARTer Pico RNA Kit (Clontech) was used for library preparation. Whole-genome sequencing was conducted using the Illumina MiSeq platform, with a read length of 60 nucleotides, producing 5,821,469 reads. FastQC^35^ was used for quality control of the data. Reads originated from Vero E6 host cells were filtered out using Bowtie 2^36^, resulting in 1,070,483 reads originated from rVSV-ΔG-spike. Mapping of the reads against the rVSV-ΔG-spike was performed using Bowtie 2 followed by variant calling using Samtools^37^, both with default parameters, resulting in a 3178× average coverage and several variants.

### Animal experiments

The animal model for SARS-CoV-2 was established by i.n. instillation of SARS-CoV-2 diluted in PBS supplemented with 2% FBS (PBF) (Biological Industries, Israel) to anesthetized [intraperitoneal ketamine (160 mg/kg) with xylazine (6 mg/kg)] 6–7-week-old golden Syrian hamsters (60–90 g, Charles River Laboratories, USA). Animals’ body weight was monitored daily. Animals were sacrificed at 3, 5, and 7 dpi for the following analyses: (1) viral load in lungs (3 and 5 dpi), (2) viral load in nasal turbinates (3 dpi), and (3) histopathological analysis (3 and 7 dpi). Viral load and histopathological procedures are described below.

Vaccination was performed by i.m. (0.05 or 0.1 ml/animal) or subcutaneous (s.c. 0.3 ml/animal) injection of rVSV-ΔG-spike to anesthetized golden Syrian hamsters (6–7 weeks old, 60–90 g). General observation for morbidity and weight loss of vaccinated animals were carried out for 7 or 11 days post vaccination. Sera was collected ~3 weeks post vaccination for titration of SARS-CoV-2 neutralizing antibodies. After 20 or 25 days post vaccination, hamsters were anesthetized, challenged i.n. with 5 × 10^6^ pfu of SARS-CoV-2, and monitored for 11–12 additional days. For isotyping of induced antibodies, C57BL/6J mice (10–14 weeks old, about 20 g) were vaccinated i.m. with 10^7^ pfu/mouse (0.1 ml/animal) of rVSV-ΔG-spike. Vaccinated mice sera were collected 14 days post vaccination to determine SARS-CoV-2 neutralizing antibodies titer and antibody isotype. All animal experiments involving SARS-CoV-2 were conducted in a BSL3 facility in accordance with the guideline of the IIBR Institutional Animal Care and Use Committee (HM-01-20, HM-02-20, HM-03-20, M-35-20).

### Lung and nasal turbinates viral load determination

Hamsters’ lungs were harvested at 3 or 5 dpi, and nasal turbinates were harvested at 3 dpi and stored at −80 °C. Lung and nasal turbinates were processed, and infectious virus quantitation was performed by plaque assay, as described above. Viral load, as well as LOD, were calculated based on volume of cell infection, dilution factor, and tissue processing volume, and presented as pfu/organ.

### Histopathology

For hematoxylin and eosin (H&E) general histopathology evaluation, lungs were rapidly isolated, and fixed in 4% neutral-buffered PFA at room temperature (RT) for 2 weeks, followed by routine processing for paraffin embedding. Coronal, serial sections, 4–5 µm thick, were performed and selected sections were stained with H&E for light microscopy examination. Images were acquired using Nikon Eclipse 50i Light Microscope (Nikon, Tokyo, Japan) or Olympus microscope (BX60)

### Histological evaluation

Lung histopathological severity score analysis was performed according to the American Thoracic Society Documents, 2011^20^. For immunolabeling of SARS-CoV-2, sections were deparaffinized and rehydrated through 100% ethanol, 95% ethanol, 70% ethanol, and 30% ethanol, washed in distilled water and antigens were retrieved using commercial antigen retrieval solution (Dako, CA, USA). Sections were then permeabilized for 10 min (0.2% Triton X-100 in PBS), blocked for 1 h (10% normal goat serum in PBS containing 0.05% Triton X-100), incubated with rabbit SARS-CoV-2 primary antibody diluted 1:200 (in-house preparation of rabbit polyclonal anti-RBD) in antibody cocktail solution (50% blocking solution, 0.05% Triton X-100 in PBS) for 24 h at 4 °C. Sections were then washed three times with washing buffer (1% blocking solution in PBS containing 0.05% Triton X-100) and incubated with anti-rabbit Alexa Fluor 488 secondary antibody (Molecular Probes, Burlington, Canada) in antibody cocktail solution for 1 h at RT. Nuclei were stained with DAPI. Following three additional washes, slides were mounted using Fluoromount-G (Southern Biotech, Al, USA) and images were acquired using a Zeiss LSM710 confocal microscope (Zeiss, Oberkochen, Germany). DAB immunohistochemical staining was performed on 4 µm formalin fixed paraffin embedded sections using Leica Bond max system (Leica Biosystems Newcastle Ltd, UK). Slides were baked for 30 min at 60 °C, dewaxed and pretreated for 5 min with epitope-retrieval solution at pH = 6 (ER1, Leica Biosystems Newcastle Ltd, UK) followed by incubation with primary antibody (in-house preparation of rabbit polyclonal anti-RBD) at 1:6000 dilution for 30 min at RT. Detection was performed using the Leica Bond Polymer Refine HRP kit (Leica Biosystems Newcastle Ltd, UK) without amplification. Briefly, a goat anti-rabbit horseradish peroxidase (HRP) polymer was applied for 10 min (RT) followed by incubation for 5 min with DAB. All slides were counterstained with hematoxylin. Morphometric analysis of DAB images was performed using MATLAB morphological-based, brightness-based, and color-based segmentation. Color segmentation of brown (cells positive for SARS-CoV-2) and blue (hematoxylin counterstaining, negative for SARS-CoV-2) was performed, and the percentage of positive cells was calculated.

### Tissue/air space ratio calculation

Tissue/air space ratio was determined using ImageJ free software analysis (particle analysis algorithm). Images of at least five random regions of interest (ROIs) per section were taken at the same magnification (×20). Color threshold parameters were determined and remained consistent throughout analysis. Total area values were measured separately for air space and tissue. Ratio of total tissue area to total air space area was calculated for each ROI. Average value of at least five ROIs per animal is presented.

### Enzyme-linked immunosorbent assay (ELISA)

Recombinant SARS-CoV-2 S glycoprotein (S2P), expressed as recently described^38^. A stabilized soluble version of the S protein (based on GenPept: QHD43416 ORF amino acids 1–1207) was designed to include proline substitutions at positions 986 and 987, and disruptive replacement of the furin cleavage site RRAR (residues at position 682–685) with GSAS. Protein expression carried out using ExpiCHO^TM^ system (Thermo Fisher Scientific, USA).

ELISA was performed as previously described^39^. Briefly, Nunc MaxiSorp ELISA plates (Thermo Fisher Scientific, USA) were coated with 100 ng/ml of S2P in carbonate bicarbonate (Sigma, Israel) at 4 °C overnight. Following standard blocking and washes, plates were incubated with HI naive or vaccinated mice sera at a dilution of 1:200 for 1 h at 37 °C. Following washes, anti-mouse IgG-, IgG1-, or IgG2c-HRP conjugates were diluted 1:2000 (for IgG) or 1:10,000 (for IgG1 and IgG2c) and used as secondary antibodies (Jackson ImmunoResearch, USA, Cat# 115-035-003, 115-035-205 lot 148255, 115-035-208 lot 146880, respectively) followed by detection with 3,5,3′,5′-tetramethylbenzidine (Millipore, USA).

### Statistical analysis

Data were analyzed with GraphPad Prism 6 software. Exact *p* values are provided for each analysis. The analyses for morbidity experiments were performed using two-tailed one unpaired *t*-test per row, with correction for multiple comparisons using Holm–Sidak method. For correlation analysis, Spearman’s correlation *r* and *p* values are indicated. Significance for viral load analysis was determined by Kruskal–Wallis test for multiple groups, and by Mann–Whitney nonparametric test for two groups. Statistical significance for isotyping analysis was determined by one-way ANOVA nonparametric test with Kruskal–Wallis test. Histological analysis statistics was determined by one-way ANOVA with Tukey’s post hoc test.

### Reporting summary

Further information on research design is available in the Nature Research Reporting Summary linked to this article.

## Supplementary information

Supplementary Information

Reporting Summary

## Data Availability

The data that support the findings of this study are available from the corresponding authors upon reasonable request. Source Data are provided with this paper.

## Source data

Source Data

## References

[1] Coronaviridae Study Group of the International Committee on Taxonomy of Viruses (2020). The species severe acute respiratory syndrome-related coronavirus: classifying 2019-nCoV and naming it SARS-CoV-2. Nat. Microbiol.

[2] Wu F (2020). A new coronavirus associated with human respiratory disease in China. Nature.

[3] Zhou P (2020). A pneumonia outbreak associated with a new coronavirus of probable bat origin. Nature.

[4] Amanat F, Krammer F (2020). SARS-CoV-2 vaccines: status report. Immunity.

[5] Hoffmann M (2020). SARS-CoV-2 cell entry depends on ACE2 and TMPRSS2 and is blocked by a clinically proven protease inhibitor. Cell.

[6] Walls AC (2020). Structure, function, and antigenicity of the SARS-CoV-2 spike glycoprotein. Cell.

[7] Hoffmann M, Kleine-Weber H, Pohlmann S (2020). A multibasic cleavage site in the spike protein of SARS-CoV-2 is essential for infection of human lung cells. Mol. Cell.

[8] Whelan, S. P. J. Vesicular stomatitis virus, in *Encyclopedia of Virology* 291–299 (Elsevier, 2008).

[9] Lawson ND (1995). Recombinant vesicular stomatitis viruses from DNA. Proc. Natl Acad. Sci. USA.

[10] Whitt MA (2010). Generation of VSV pseudotypes using recombinant DeltaG-VSV for studies on virus entry, identification of entry inhibitors, and immune responses to vaccines. J. Virol. Methods.

[11] Rodriguez SE (2019). Vesicular stomatitis virus-based vaccine protects mice against Crimean-Congo hemorrhagic fever. Sci. Rep..

[12] Suder E (2018). The vesicular stomatitis virus-based Ebola virus vaccine: from concept to clinical trials. Hum. Vaccin Immunother..

[13] Monath TP (2019). rVSVDeltaG-ZEBOV-GP (also designated V920) recombinant vesicular stomatitis virus pseudotyped with Ebola Zaire glycoprotein: standardized template with key considerations for a risk/benefit assessment. Vaccine X.

[14] Fathi A, Dahlke C, Addo MM (2019). Recombinant vesicular stomatitis virus vector vaccines for WHO blueprint priority pathogens. Hum. Vaccines Immunother..

[15] Thanh Le,T (2020). The COVID-19 vaccine development landscape. Nat. Rev. Drug Discov..

[16] Liu K (2020). Clinical characteristics of novel coronavirus cases in tertiary hospitals in Hubei Province. Chin. Med. J..

[17] Chan JF (2020). Simulation of the clinical and pathological manifestations of Coronavirus Disease 2019 (COVID-19) in golden Syrian hamster model: implications for disease pathogenesis and transmissibility. Clin. Infect. Dis..

[18] Imai M (2020). Syrian hamsters as a small animal model for SARS-CoV-2 infection and countermeasure development. Proc. Natl Acad. Sci. USA.

[19] Osterrieder N (2020). Age-dependent progression of SARS-CoV-2 infection in Syrian Hamsters. Viruses.

[20] Matute-Bello G (2011). An official American Thoracic Society workshop report: features and measurements of experimental acute lung injury in animals. Am. J. Respir. Cell Mol. Biol..

[21] Ujike M (2016). The contribution of the cytoplasmic retrieval signal of severe acute respiratory syndrome coronavirus to intracellular accumulation of S proteins and incorporation of S protein into virus-like particles. J. Gen. Virol..

[22] Fukushi S (2005). Vesicular stomatitis virus pseudotyped with severe acute respiratory syndrome coronavirus spike protein. J. Gen. Virol..

[23] Kapadia SU (2005). Long-term protection from SARS coronavirus infection conferred by a single immunization with an attenuated VSV-based vaccine. Virology.

[24] Ou X (2020). Characterization of spike glycoprotein of SARS-CoV-2 on virus entry and its immune cross-reactivity with SARS-CoV. Nat. Commun..

[25] Case, J. B. et al. *Neutralizing Antibody and Soluble ACE2 Inhibition of a Replication-Competent VSV-SARS-CoV-2 and a Clinical Isolate of SARS-CoV-2*. 10.1101/2020.05.18.102038 (2020). 10.1016/j.chom.2020.06.021 PMC7332453 32735849

[26] Dieterle, M. E. et al. *A Replication-Competent Vesicular Stomatitis Virus for Studies of SARS-CoV-2 Spike-Mediated Cell Entry and its Inhibition*. 10.1101/2020.05.20.105247 (2020). 10.1016/j.chom.2020.06.020 PMC7332447 32738193

[27] Lau SY (2020). Attenuated SARS-CoV-2 variants with deletions at the S1/S2 junction. Emerg. Microbes Infect..

[28] Ols S (2020). Route of vaccine administration alters antigen trafficking but not innate or adaptive immunity. Cell Rep..

[29] Zuckerman JN (2000). The importance of injecting vaccines into muscle. Different patients need different needle sizes. BMJ.

[30] Zhang L, Wang W, Wang S (2015). Effect of vaccine administration modality on immunogenicity and efficacy. Expert Rev. Vaccines.

[31] Sia SF (2020). Pathogenesis and transmission of SARS-CoV-2 in golden hamsters. Nature.

[32] Bottazzi ME (2020). Coronavirus vaccine-associated lung immunopathology-what is the significance?. Microbes Infect..

[33] Case JB (2020). Replication-competent vesicular stomatitis virus vaccine vector protects against SARS-CoV-2-mediated pathogenesis in mice. Cell Host Microbe.

[34] Maor Y (2020). Compassionate use of convalescent plasma for treatment of moderate and severe pneumonia in COVID-19 patients and association with IgG antibody levels in donated plasma. EClinicalMedicine.

[35] Bittencourt, S. A. *FastQC: a Quality Control Tool for High Throughput Sequence Data*. https://www.bioinformatics.babraham.ac.uk/projects/fastqc/ (2010).

[36] Langmead B, Salzberg SL (2012). Fast gapped-read alignment with Bowtie 2. Nat. Methods.

[37] Li H (2009). The sequence alignment/map format and SAMtools. Bioinformatics.

[38] Noy-Porat T (2020). A panel of human neutralizing mAbs targeting SARS-CoV-2 spike at multiple epitopes. Nat. Commun..

[39] Israely T (2014). TLR3 and TLR9 agonists improve postexposure vaccination efficacy of live smallpox vaccines. PLoS ONE.

