# Supplementary material for: A single dose of recombinant VSV-∆G-spike vaccine provides protection against SARS-CoV-2 challenge
Source: Nat Commun. 2020 Dec 16;11:6402. doi: 10.1038/s41467-020-20228-7 (PMC7745033; doi:10.1038/s41467-020-20228-7)
Supplement: Supplementary file 1 — Supplementary Information [file 41467_2020_20228_MOESM1_ESM.pdf]

Supplementary Information for:

A single dose of recombinant VSV-∆G-spike vaccine provides protection against SARS-CoV-2 challenge

Yfat Yahalom-Ronen*, Hadas Tamir*, Sharon Melamed*, Boaz Politi*, Ohad Shifman*, Hagit Achdout, Einat B. Vitner, Ofir Israeli, Elad Milrot, Dana Stein, Inbar Cohen-Gihon, Shlomi Lazar, Hila Gutman, Itai Glinert, Lilach Cherry, Yaron Vagima, Shirley Lazar, Shay Weiss, Amir Ben-Shmuel, Roy Avraham, Reut Puni, Edith Lupu, Elad Bar David, Assa Sittner, Noam Erez, Ran Zichel, Emanuelle Mamroud, Ohad Mazor, Haim Levy, Orly Laskar, Shmuel Yitzhaki, Shmuel C. Shapira, Anat Zvi, Adi Beth-Din, Nir Paran^ and Tomer Israely^

Supplementary Figure 1:

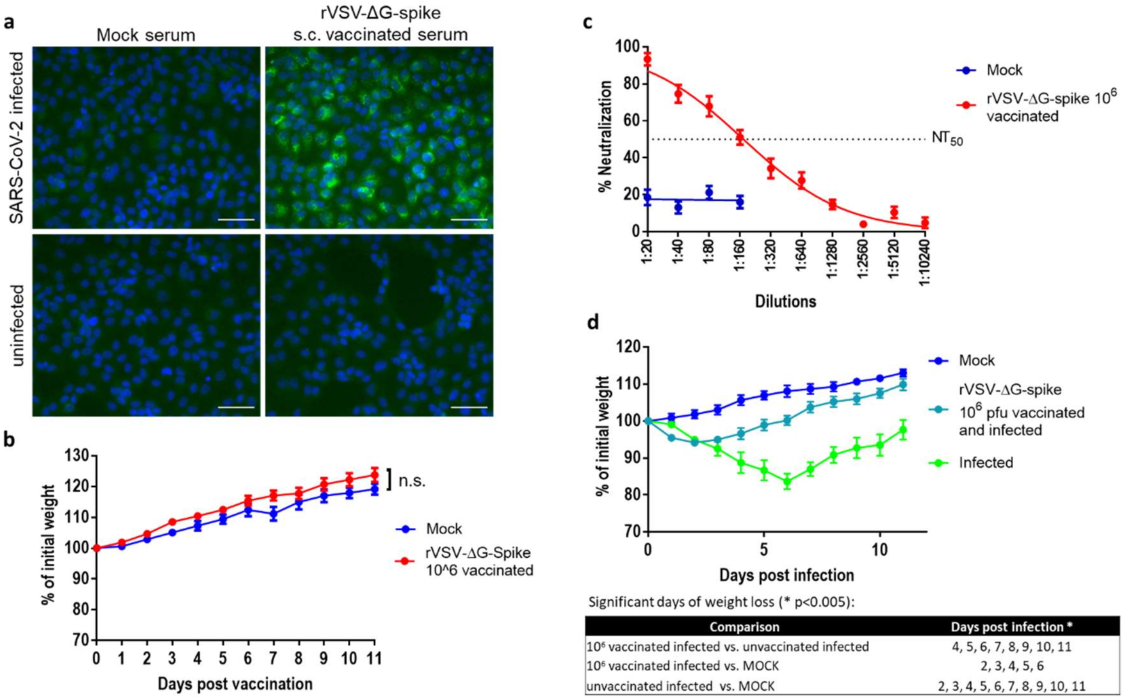

Comparison	Days post infection *
10^6 vaccinated infected vs. unvaccinated infected	4, 5, 6, 7, 8, 9, 10, 11
10^6 vaccinated infected vs. MOCK	2, 3, 4, 5, 6
unvaccinated infected vs. MOCK	2, 3, 4, 5, 6, 7, 8, 9, 10, 11

Supplementary Figure 1. A single-dose s.c. rVSV-ΔG-spike vaccine safety and efficacy in hamsters following SARS-CoV-2 challenge. a Immunofluorescent images of Vero E6 cells infected with SARS-CoV-2, stained with sera from either mock-vaccinated hamsters (left panel) or rVSV-ΔG-spike vaccinated-hamsters (right panel). Representative images of three experiments are presented. Scale bars: 50 µm. **b** Body weight changes of naïve hamsters (Mock, n=4; these animals also served as the Mock group in Fig. 4a), and hamsters vaccinated s.c. with rVSV-ΔG-spike (n=16 in days 0-7, n=12 in days 8-12). Statistical analysis was performed using two-tailed one unpaired t-test per row with correction for multiple comparisons using Holm-Sidak method. No statistical difference was observed (n.s.). **c** PRNT of hamster sera collected from mock-vaccinated hamsters (n=5) or hamsters vaccinated s.c. with rVSV-ΔG-spike (n=5). **d** Body weight changes of hamsters infected with SARS-CoV-2 (n=4), and hamsters vaccinated and infected with SARS-CoV-2 ($5x10^6$ pfu/hamster, n=14) 25 days post-vaccination, compared to mock hamsters (n=4). Three hamsters from vaccinated and infected group, and one hamster from infected group, were sacrificed at 5dpi for additional analyses. Statistical significance was determined by two-tailed one unpaired t-test per row, with correction for multiple comparisons using Holm-Sidak method. * p<0.005. Data for (**b**, **c**, **d**) are presented as mean values ± SEM. Source data are provided as a Source Data file.

Supplementary Figure 2:

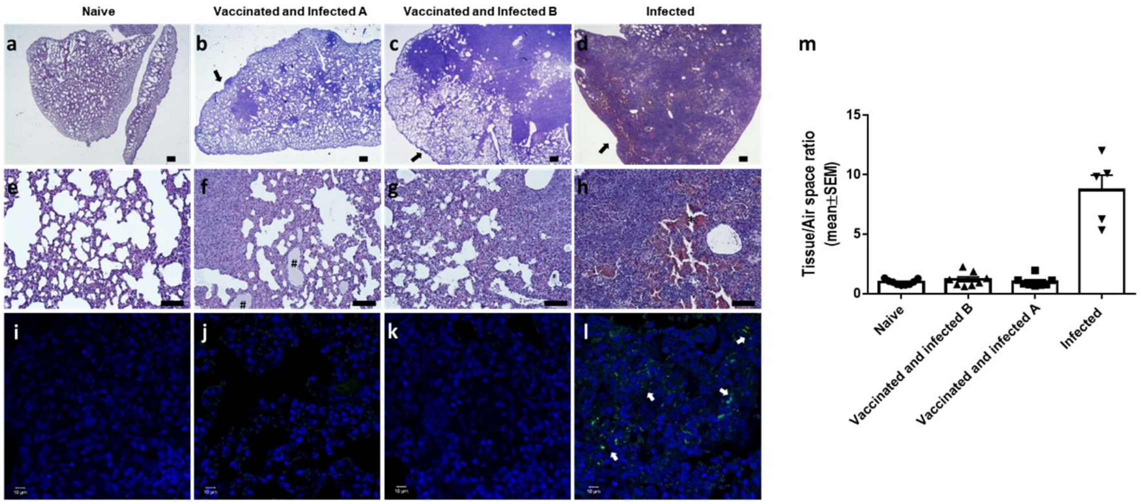

Supplementary Figure 2. Histopathological analysis of SARS-CoV-2 infected lungs following s.c. rVSV-ΔG-spike vaccination. General histology (H&E) and SARS-CoV-2 Immunolabeling of naïve, unvaccinated infected ($5x10^6$), and vaccinated and infected (10^6 pfu) hamsters' lungs. Lungs were isolated and processed for paraffin embedding from naïve (**a**, **e**, **i**), vaccinated and infected (**b**, **f**, **j**, **c**, **g**, **k**), and infected ($5x10^6$ pfu) (**d**, **h**, **l**) 5 dpi. Sections (5 µm) were taken for H&E staining (**a**-**h**) and SARS-CoV-2 immunolabeling (**i**-**l**, DAPI-Blue, SARS-CoV-2-Green). Images **a**-**d**: bar= 100µm; images **e**-**h**: bar= 100µm; images **i**-**l**: bar= 10µm. Black arrows indicate patches of focal inflammation, pleural invagination and alveolar collapse. "*"- indicates hemorrhagic areas. "#"- indicates edema and protein rich exudates. Black arrow heads indicate pulmonary mononuclear cells. White arrows indicate SARS-CoV-2 positive staining. **m** Tissue/Air space ratio. Each column represents one hamster. The data were analyzed from at least 5ROIs per animal, and is presented in bars as mean ± SEM. Naïve group: n=1, vaccinated and infected: n=2, infected: n=1. Source data are provided as a Source Data file.
