# Supplementary material for: A single dose of recombinant VSV-∆G-spike vaccine provides protection against SARS-CoV-2 challenge
Source: Nat Commun. 2020 Dec 16;11:6402. doi: 10.1038/s41467-020-20228-7 (PMC7745033; doi:10.1038/s41467-020-20228-7)
Supplement: Supplementary file 2 — Reporting Summary [file 41467_2020_20228_MOESM2_ESM.pdf]

nature research

Corresponding author(s): Israely Tomer, Paran Nir

Last updated by author(s): Nov 4, 2020

Reporting Summary

Nature Research wishes to improve the reproducibility of the work that we publish. This form provides structure for consistency and transparency in reporting. For further information on Nature Research policies, see our Editorial Policies and the Editorial Policy Checklist.

Statistics

For all statistical analyses, confirm that the following items are present in the figure legend, table legend, main text, or Methods section.

n/a	Confirmed	
☐	☒	The exact sample size (n) for each experimental group/condition, given as a discrete number and unit of measurement
☒	☐	A statement on whether measurements were taken from distinct samples or whether the same sample was measured repeatedly
☐	☒	The statistical test(s) used AND whether they are one- or two-sided<br>*Only common tests should be described solely by name; describe more complex techniques in the Methods section.*
☒	☐	A description of all covariates tested
☐	☒	A description of any assumptions or corrections, such as tests of normality and adjustment for multiple comparisons
☐	☒	A full description of the statistical parameters including central tendency (e.g. means) or other basic estimates (e.g. regression coefficient) AND variation (e.g. standard deviation) or associated estimates of uncertainty (e.g. confidence intervals)
☐	☒	For null hypothesis testing, the test statistic (e.g. F, t, r) with confidence intervals, effect sizes, degrees of freedom and P value noted<br>*Give P values as exact values whenever suitable.*
☒	☐	For Bayesian analysis, information on the choice of priors and Markov chain Monte Carlo settings
☒	☐	For hierarchical and complex designs, identification of the appropriate level for tests and full reporting of outcomes
☒	☐	Estimates of effect sizes (e.g. Cohen's d, Pearson's r), indicating how they were calculated

Our web collection on statistics for biologists contains articles on many of the points above.

Software and code

Policy information about availability of computer code

Data collection	N/A
Data analysis	Graphs and statistical analyses were performed in GraphPad Prism 6, Microsoft Excel 2016, ImageJ, Adobe Photoshop, and MATLAB. Figures were prepared using GraphPad Prism 6 and Power point 2016.

For manuscripts utilizing custom algorithms or software that are central to the research but not yet described in published literature, software must be made available to editors and reviewers. We strongly encourage code deposition in a community repository (e.g. GitHub). See the Nature Research guidelines for submitting code & software for further information.

Data

Policy information about availability of data

All manuscripts must include a data availability statement. This statement should provide the following information, where applicable:

- Accession codes, unique identifiers, or web links for publicly available datasets
- A list of figures that have associated raw data
- A description of any restrictions on data availability

The data that support the findings of this study are available from the corresponding author upon reasonable request.
Figure 1: Plasmid sequences will be available upon request under an MTA.

Field-specific reporting

Please select the one below that is the best fit for your research. If you are not sure, read the appropriate sections before making your selection.

- [x] Life sciences
- [ ] Behavioural & social sciences
- [ ] Ecological, evolutionary & environmental sciences

For a reference copy of the document with all sections, see nature.com/documents/nr-reporting-summary-flat.pdf

Life sciences study design

All studies must disclose on these points even when the disclosure is negative.

Sample size	In this study sample size was selected without pre-calculation. This was done due to the complete lack of previous data concerning the expected diversity in individual animal responses, as well as the group variation in results. Based on our extensive previous experience with other viral pathogens, we chose groups of 4-16 animals for morbidity and protection analysis, which allows reliable statistical analysis post-experiment.<br>In vitro studies were performed with replicates and repeated several times based on our previous experience.<br>Citation: Israely T, Melamed S, Achdout H, Erez N, Politi B, Waner T, et al. (2014) TLR3 and TLR9 Agonists Improve Postexposure Vaccination Efficacy of Live Smallpox Vaccines. PLoS ONE 9(10): e110545. https://doi.org/10.1371/journal.pone.0110545.<br>Melamed, S., Avraham, R., Rothbard, D.E. et al. Innate immune response in neuronopathic forms of Gaucher disease confers resistance against viral-induced encephalitis. acta neuropathol commun 8, 144 (2020). https://doi.org/10.1186/s40478-020-01020-6.
Data exclusions	No data was excluded.
Replication	Safety and efficacy experiments as well as IFA and EM were replicated independently more than 3 times with similar results as indicated in the figure legends. Histopathological analysis was performed on 2 independent vaccine lots showing similar results.
Randomization	All animals were randomly assigned to the experimental groups. For in-vitro study, randomization is irrelevant.
Blinding	In our experience, preliminary animal studies, especially performed under BSL-3 conditions, do not require blinding and thus were not blinded in this case. The identity of each group was essential in the performance of the experiments.

Reporting for specific materials, systems and methods

We require information from authors about some types of materials, experimental systems and methods used in many studies. Here, indicate whether each material, system or method listed is relevant to your study. If you are not sure if a list item applies to your research, read the appropriate section before selecting a response.

Materials & experimental systems

n/a	Involved in the study	
[ ]	[x]	Antibodies
[ ]	[x]	Eukaryotic cell lines
[x]	[ ]	Palaeontology and archaeology
[ ]	[x]	Animals and other organisms
[ ]	[x]	Human research participants
[x]	[ ]	Clinical data
[x]	[ ]	Dual use research of concern

Methods

n/a	Involved in the study	
[x]	[ ]	ChIP-seq
[x]	[ ]	Flow cytometry
[x]	[ ]	MRI-based neuroimaging

Antibodies

Antibodies used	Anti-mouse IgG-horseradish peroxidase (HRP) Jackson ImmunoResearch, USA Cat# 115-035-003 1:2000; IgG1 horseradish peroxidase (HRP) Jackson ImmunoResearch, USA Cat# 115-035-205 lot#148255 1:10000; IgG2c-horseradish peroxidase (HRP) Jackson ImmunoResearch, USA Cat# 115-035-208 lot#146880 1:10000; alexa-fluor goat anti rabbit FITC sigma Cat#F6005 lot#107K6086 1:200; Anti rabbit IgG gold secondary ab. Sigma Cat# G3779 lot# SLBR2479V 1:20; pAB RBD SinoBiological Cat#SBF40150-T62 lot#HD11SE1501 1:30; hyperimmune rabbit in-house prep 1:200; anti-RBD in-house prep 1:200; alexa-fluor 488 conjugated goat anti-hamster Jackson ImmunoResearch, USA Cat# 107-545-142 lot#147500 1:100; alexa-fluor FITC conjugated goat anti-human Sigma Cat# F0132 lot#SLBR4951V 1:200; Naïve/ vaccinated hamsters sera in-house prep 1:200.
Validation	Primary antibodies specificity was validated by staining SARS-CoV-2 infected cells in parallel to mock infected cells. Staining was specific to infected cells, whereas no staining was observed in mock infected cells. Secondary antibodies specificity was validated by staining with the secondary antibody without primary antibody, and showing no signal.

Eukaryotic cell lines

Policy information about cell lines

Cell line source(s)	Vero E6, BHK-21, both from ATCC.
Authentication	Authentications were performed according to characteristics features as described by ATCC.
Mycoplasma contamination	Cell lines were tested negative for Mycoplasma.
Commonly misidentified lines (See ICLAC register)	No commonly misidentified cell lines were used in the study.

Animals and other organisms

Policy information about studies involving animals; ARRIVE guidelines recommended for reporting animal research

Laboratory animals	6-7 weeks old female golden Syrian hamsters. 10-14 weeks female C57BL/6J mice. animals were housed in IVC cages fitted with solid bottom and filled with certified commercial wood shaving and bedding material. All animals maintained at constant room temperature 20 - 24 °C and 30 - 70% relative humidity with a 12-h light/12-h dark photo-period.
Wild animals	The study did not involve wild animals.
Field-collected samples	The study did not involve samples collected in the field.
Ethics oversight	All animal experiments involving SARS-CoV-2 were conducted in a BSL3 facility in accordance with the guideline of the Israel Institute for Biological Research (IIBR) animal experiments committee. Protocol numbers: #HM-01-20, HM-02-20, HM-03-20, M-35-20.

Note that full information on the approval of the study protocol must also be provided in the manuscript.

Human research participants

Policy information about studies involving human research participants

Population characteristics	N/A
Recruitment	Sera from 12 convalescent COVID-19 patients was collected by The National Blood services of "Magen David Adom" in Israel for research purposes. All donors gave their informed consent to the donation.
Ethics oversight	N/A

Note that full information on the approval of the study protocol must also be provided in the manuscript.
